# Long-Term Balance Outcomes in Vestibular Ablative Surgeries

**DOI:** 10.4274/tao.2020.6032

**Published:** 2021-03-26

**Authors:** Fakih Cihat Eravcı, Metin Yılmaz, Ebru Şansal, Nagihan Gülhan, Recep Karamert, Hakan Tutar, Mehmet Birol Uğur

**Affiliations:** 1 Department of Otorhinolaryngology, Gazi University Faculty of Medicine, Ankara, Turkey; 2 Department of Audiology, Gazi University Faculty of Medicine, Ankara, Turkey

**Keywords:** Ménière’s disease, vestibular nerve, labyrinth, surgery, postural balance, dizziness, disability evaluation

## Abstract

**Objective:**

To evaluate the long-term balance outcomes of vestibular nerve section (VNS) and labyrinthectomy (L) operations. The indirect outcomes will be the correlation of objective and subjective test results and an analysis of anterior-posterior versus medial-lateral computerized posturography (CP) scores.

**Methods:**

This retrospective study evaluated objective CP and subjective Dizziness Handicap Inventory (DHI) results of patients who underwent VNS and L surgeries for Ménière’s disease.

**Results:**

A total of 55 (31 VNS and 24 L) patients were included in the study. The two operation groups were similar in terms of age, and mean time between surgery and the tests (p=0.465 and p=0.616) respectively. The vestibular and global scores at anterior-posterior CP showed statistically significant differences between the groups (p=0.000 and p=0.007) respectively in favor of the VNS group. In addition, the comparison of the vestibular CP scores of anterior-posterior and medial-lateral evaluations of the entire study population was lower in the medial-lateral evaluation (p=0.000). The mean DHI scores did not show statistically significant differences (p=0.359) between operation groups, nor did the correlation analysis between CP and DHI scores reveal statistical significance (p values >0.05).

**Conclusion:**

In the long term, objective balance outcomes are better for VNS patients than for L patients. Additionally, medial-lateral balance outcomes are more affected than anterior-posterior balance outcomes from unilateral ablative surgeries. Subjective balance perception is not different between the two surgery groups, and DHI scores do not show a correlation with CP scores.

## Introduction

Ménière’s disease (MD) presents as recurrent episodic vertigo attacks, aural fullness, tinnitus, and sensorineural hearing loss generally accompanies the clinical situation. Although it has a low incidence, it can affect the patient’s quality of life and results in serious consequences (1). The strategy for treatment is a stepped approach starting with more conservative measures, such as dietary modification and oral medication. It is reported that these treatment options are sufficient for 60% to 87% of the patients to maintain their daily activities (2). The next treatment step for patients with ongoing disabling attacks despite conservative treatments are minimally invasive options, such as intratympanic injections and endolymphatic sac decompression. The most successful options, however, are the most invasive—vestibular nerve section (VNS) and labyrinthectomy (L). These ablative surgical options have resulted in over 90% vertigo control in patients with refractory MD (3, 4). Yet, these ablative surgeries result in a unilateral loss of vestibular function, and their actual long-term success depends on sufficient vestibular compensation as impaired vestibular compensation can result in postural instability.

These two ablative surgeries were introduced over 100 years ago and are still being performed due to their success in controlling vertigo attacks (5). Although these operations have morbidities, these are far outweighed by the life-threatening Tumarkin attacks and the clinical presentations in which patients cannot maintain daily activities. Choosing between these two surgical options for relief from severe vertigo attacks depends on the hearing level of the patient and perioperative morbidity risks. Disequilibrium is the main complaint in the long term of patients who have undergone ablative surgeries. If there is a difference in terms of postoperative unsteadiness between these surgeries, it may provide another parameter for deciding on the procedure. Previous studies on vestibular ablative surgeries for MD have generally focused on the success of controlling vertigo attacks; only a few have addressed the balance outcomes of the procedures (6). Unsteadiness after the operation has been reported more frequently from L, at rates ranging from 20% to 28% and with 14% to 20% being after VNS operations (7, 8). A study searching the long-term balance outcomes of both VNS and L surgeries reported a high incidence of incomplete vestibular compensation that did not result in a significant balance handicap, and found no difference in terms of balance between the operations (9). However, another study showed that the L group complained more from subjective dizziness (7). Research on this topic is nearly non-existent, so the controversy continues.

In the presented study, we aimed to evaluate the differences in objective and subjective balance outcomes with computerized posturography (CP) and the Dizziness Handicap Inventory (DHI) in patients who underwent VNS and L procedures. The second outcome will be the chance to explore the relationship between CP and DHI.

## Methods

The presented study was conducted in the Department of Otorhinolaryngology, Gazi University Hospital, Ankara, Turkey, approved by the local ethics committee (approval no: 01-09/2019), and carried out in accordance with the principles of the Declaration of Helsinki. All subjects were informed, and consent forms were obtained preoperatively. This retrospective study evaluated the demographic data, CP, and DHI results of the patients that underwent retro-sigmoid VNS and trans-mastoid L for MD between 2008 and 2019. The study criteria included patients aged 18 years and above who underwent one of the mentioned surgical procedures, followed by CP and DHI after one year postoperatively. The criteria excluded patients who had bilateral MD (one patient), central neural system pathology (one patient), those who had not been tested postoperatively (12 patients), and those who had been tested earlier than one year (two patients). Based on these criteria 31 VNS and 24 L patients were included in the study.

### Computerized Posturography

The CP used in this study was from Synapsys Posturography Systems (Version 3.0, Marseille, France). The test was administered by an experienced audiologist. The results of the patients’ ability to use inputs from vestibular, visual, and proprioceptive systems to control balance were obtained with the sensory organization test (SOT) in a numeric and easily interpretable graph.

The SOT protocols consist of six steps (conditions) assessed separately for anterior-posterior and medial-lateral evaluation and applied in the order of simple to forcible. The platform was static during the first three conditions: eyes were open in the first, closed in the second (Romberg), and the third was applied with a visual environment that was synchronized with postural fluctuation of the patient. The second group of three conditions were performed on a dynamic platform: eyes were open in the fourth, closed in the fifth, and with visual environment in the sixth condition.

Sensory analysis is used to evaluate the loss of function in sensory perception by proportioning the average equilibrium scores in relation to each other. There are five scores: somatosensorial score (condition 1 ÷ condition 2), visual score (condition 4 ÷ condition 1), vestibular score (condition 5 ÷ condition 1), preferential score (condition 3 + condition 6 ÷ condition 2 + condition 5) and global score (overall score incorporating all conditions). In our study, we focused on vestibular score since the somatosensory system is eliminated by a moving platform and the visual system is eliminated with eyes closed. Therefore, this score indicates the patient’s ability to use vestibular inputs.

### Dizziness Handicap Inventory

DHI is an inventory used to determine the changes in the quality of life in dizzy patients. The Turkish version of the DHI was applied to all patients (10). The inventory comprises 25 statements that investigate the conditions in daily activities, that are answered with “*yes”,* “*sometimes”* or “*no”*. These answers are scored as 4, 2, and 0, respectively, and total DHI scores (ranging from 0 to 100) are obtained by summing all scores. Higher scores indicate greater dizziness handicap.

### Statistical Analysis

Statistical analyses were performed using the SPSS software (SPSS version 22, Armonk, NY, USA). The results are presented as the mean ± standard deviation (minimum-maximum). The one-sample Kolmogorov-Smirnov test was used to determine the normality. Because this test showed normal distribution for all data, parametric tests were used. The study population was classified according to the two operations, and differences in gender and the side that was operated on were assessed using the chi-square Test. The differences in CP and DHI scores were assessed using the student’s two-tailed t-test. Additionally, all CP scores from anterior-posterior and medial-lateral evaluations were compared for the entire study population with the paired t-test. A Pearson’s correlation coefficient test was used for correlation analysis between the CP and DHI scores for the entire study population and related correlation coefficient (r) values were shared. In all statistical analyses, a p-value of <0.05 was considered significant.

## Results

A total of 55 patients with a mean age of 44.1±12.3 (18–75) years were included in the study. Thirty-one patients had undergone retro-sigmoid VNS and 24 had undergone trans-mastoid L. The mean time between surgery and testing was 3.3±1.5 (1–7) years. The demographic results for each group are summarized in Table 1. The two groups were similar in terms of age, operated side and the mean time between surgery and tests (p=0.465, p=0.464, and p=0.616, respectively). Gender analysis, however, revealed statistical significance (p=0.003).

**Figure 1:**
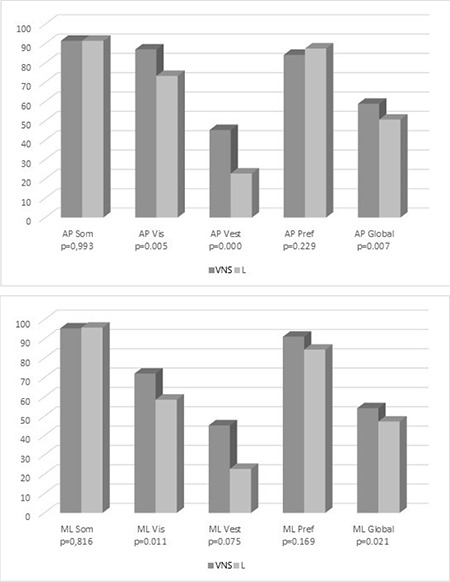
Mean computerized posturography (CP) scores for vestibular nerve section (VNS) and labyrinthectomy (L) group AP: Anterior-posterior evaluations, ML: Medial-lateral evaluations, Som: Somatosensorial score, Vis: Visual score, Vest: Vestibular score, Pref: Preferential score, Global: Global score

The mean CP scores in the anterior-posterior evaluation are somatosensorial score, visual score, vestibular score, preferential score, and global score; for the VNS group, they were 91.5±10.0 (58–100), 87.0±11.3 (44–100), 45.3±23.9 (0–83), 84.2±11.1 (66–100), and 58.9±11.3 (28–78), respectively. The CP scores in the anterior-posterior evaluation, in the same order, for the L group were 91.5±11.5 (58–100), 73.3±19.7 (22–96), 22.8±17.1 (0–58), 87.5±11.3 (66–100), and 50.7±10.2 (28–64), respectively. Likewise, the CP scores in medial-lateral evaluation representing the somatosensorial score, visual score, vestibular score, preferential score, and global score for the VNS group were 95.5±6.1 (68–100), 72.1±17.1 (0–91), 17.0±20.2 (0–56), 91.2±15.6 (21–100), and 54.2±11.7 (20–71), respectively. The CP scores in medial-lateral evaluation, in the same order, for the L group were 95.9±6.7 (68–100), 58.6±19.6 (0–80), 8.5±14.2 (0–50), 84.5±19.3 (21–100), and 47.3±9.8 (20–62). These scores are shown in Figure 1. The visual score, vestibular score, and global score from the anterior-posterior evaluation showed statistically significant differences between the groups (p=0.005, p=0.000, and p=0.007, respectively) in favor of the VNS group. Similarly, the visual score, vestibular score, and global score from the medial-lateral evaluation resulted in p=0.011, p=0.075, and p=0.021, respectively, in the statistical analyses, again in favor of the VNS group.

The comparison of the CP scores of anterior-posterior and medial-lateral scores of the entire study population is summarized in Table 2. The vestibular scores were lower at medial-lateral evaluation (p<0.001).

**Figure 2:**
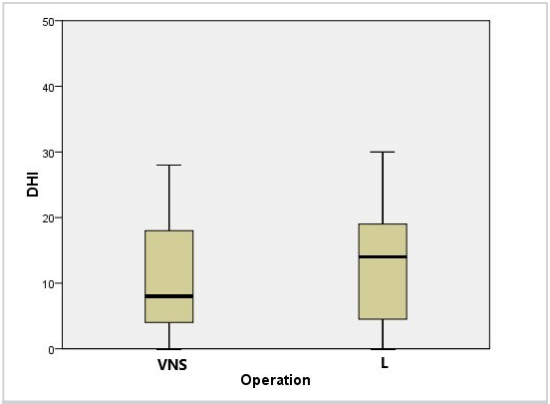
Dizziness Handicap Inventory (DHI) scores for vestibular nerve section (VNS) and labyrinthectomy (L) groups

The mean DHI scores of the VNS and L operation groups were 11.4±10.6 (0–44) and 14.6±14.0 (0–50), respectively. These are shown in Figure 2. The DHI score analysis between the two operations did not show any statistical difference (p=0.359).

The correlation analysis of DHI scores with vestibular scores and global scores did not show any statistically significance (p=0.252, r=-0.157; p=0.100, r=-0.224 for anterior-posterior and p=0.303 r=-0.141, p=0.186, r=-0.181 for medial-lateral evaluations, respectively).

## Discussion

Vestibular ablative surgeries are performed to control the severe vertigo attacks of MD that are intractable to conservative treatment options. These procedures have been performed for over a century. They are reliable and a great deal of experience has been accumulated. Although these procedures are invasive and destructive, recovering from attacks that threaten life and severely disrupt patients’ quality of life outweighs the nature of the procedures. In the recent decades, intratympanic aminoglycosides attracted attention for MD treatment as this intervention is a targeted treatment directly to the inner ear with a minimally invasive method and minimal systemic side effects (11). On the other hand, the toxicity of aminoglycosides and lower vertigo control, compared with ablative surgeries, are the chief concerns. Despite being referred to as a destructive procedure, VNS is a hearing-preserving operation. Moreover, in the long term for MD patients, vestibular compensation is determinative of success and can be accomplished in the case of a stabilized vestibular system that rescues the central nervous system from fluctuating vestibular signals, but intratympanic aminoglycoside applications cannot ensure this process because the signals from the inner ear are not totally eliminated. Accordingly, ablative surgeries suggest better vestibular compensation, but unfortunately, this topic requires new studies and elucidation. Even studies about vestibular compensation of ablative surgeries and comparisons between them are scarce. Therefore, in the presented study, we evaluated the objective CP and subjective DHI scores of VNS and L patients. The results revealed that while the subjective DHI scores did not differ between the operations, the VNS group showed better results, confirmed by CP, in objective vestibular and global scores.

Vestibular compensation is a multifactorial process in which age, postoperative time, and surgical differences can affect the results. In the presented study, the two different ablative surgical procedure groups were similar in terms of age and operative time, and therefore we focused on the effect of surgical features on balance outcomes. In theory, there are four different allegations about the differences between VNS and L that can impact the vestibular compensation process. First, L is a preganglionic deafferentation procedure in which ablation is performed peripheral to the ganglion. This is a controversial topic as some allege that this preservation of ganglion plays a role in spontaneous vestibular activity and contributes to the vestibular compensation process (12). Other studies have proposed that this activity may lead to a failure of vertigo control (13). The ambiguity in this issue is whether the ganglion cells survive in the long term or if they later grow as fibrous tissue forming a traumatic neuroma (5). Therefore, the negative or positive impact of ganglion preservation and spontaneous vestibular activity on vestibular compensation is not clear. On the other hand, a total VNS is quite difficult with the retro-labyrinthine approach due to variations in acousticofacial bundle anatomy, and again there is controversy about retained vestibular fibers. Some studies attribute the 10% failure of vertigo control from VNS to these fibers, others speculate that this can contribute to the vestibular compensation process (3, 9, 14).

The other two concerns about retro-sigmoid VNS are that-because it is an intradural procedure-it may delay postoperative mobilization, and retraction of the cerebellum may have a negative effect on vestibular compensation. In our clinic, however, we mobilize patients with VNS as soon as possible, and generally, these patients do not differ from L patients in terms of mobilization time. Additionally, we do not use a cerebellum retractor, rather, we retract delicately with minor surgical instruments until opening the cistern and drainage of the cerebrospinal fluid. These two precautions may have contributed to our VNS results. As we stated, the most common studies related to ablative vestibular surgeries are mainly focused on vertigo control rates. Apart from these, the scarce studies that have focused on vestibular compensation have revealed that there was no difference in terms of disequilibrium between VNS and L, according to self-reported dizziness surveys and posturography performance (9). In contrast, another study revealed that their L group complained more of subjective dizziness, and they attributed this result to the advanced age of the L group (7). In our study, however, we found that the L group’s CP scores on the vestibular and global components were lower compared to the VNS group, despite both groups being similar in terms of age. This may be related to the minimal vestibular function in the retained fibers due to incomplete VNS or upregulated proprioceptive inputs (15). Additionally, in our study, thanks to the CP feature, we had the chance to evaluate balance outcomes from anterior-posterior and medial-lateral separately, and to the best of our knowledge, this is the first study finding that medial-lateral motions provoke dizziness more severely in vestibular ablative surgeries.

In contrast to the objective parameters, we did not find a statistical difference in subjective DHI scores between the surgical groups. This subjective dizziness perception is probably affected also by emotional and individual characteristics (16). Additionally, the correlation of objective CP scores and subjective DHI scores are a controversial issue—some studies have found a moderate relation and others found no relation (17, 18). In our study, we did not find statistical significance and considered the results objective; subjective tests do not always correlate with each other since perceptions can vary between individuals.

Studies and evidence on this topic remain scarce, and to the best of our knowledge, the number we reached is the highest in the literature related to ablative surgeries that have been assessed by both objective and subjective tests together. Nevertheless, the study has certain limitations. The retrospective nature of the study hinders gathering additional information, such as the mean mobilization time after surgery, and applying a broad range of objective vestibular tests and establishing the correlation between the two.

## Conclusion

In conclusion, the presented study’s results show that objective balance outcomes in the long term seem better from VNS than from L ablative surgeries. Further, medial-lateral balance outcomes are more affected than anterior-posterior balance outcomes by unilateral ablative surgeries. Subjective balance perception is not different between the two, and subjective DHI scores do not show a correlation with objective CP scores.

**Main Points**

• The most successful surgical treatment options in patients with refractory Ménière’s disease are vestibular nerve section (VNS) and labyrinthectomy (L).

• These ablative surgeries result in a unilateral loss of vestibular function, and their actual long-term success depends on sufficient vestibular compensation.

• In the presented study, we evaluated the patients with computerized posturography and the dizziness handicap inventory (DHI).

• Results revealed that the VNS group showed better results in objective vestibular and global scores at computerized posturography. However, subjective DHI scores were similar.

• Additionally, computerized posturography results revealed that medial-lateral motions provoke dizziness more severely than anterior-posterior motions in vestibular ablative surgeries.

## Figures and Tables

**Table 1:**
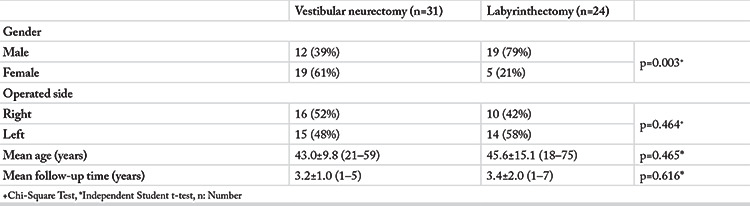
Demographic results of study groups

**Table 2:**
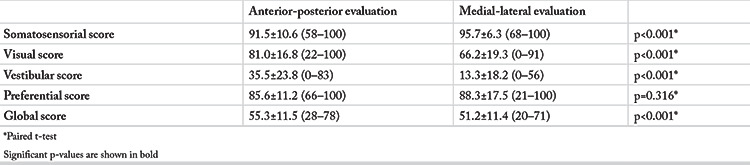
Computerized posturography (CP) scores in anterior-posterior and medial-lateral evaluations in entire study population
